# Association of CD4-positive cell infiltration with response to vedolizumab in patients with ulcerative colitis

**DOI:** 10.1038/s41598-023-47618-3

**Published:** 2023-11-20

**Authors:** Haruka Miyazaki, Namiko Hoshi, Tsukasa Ishida, Chiharu Nishioka, Sachiko Ouchi, Daisuke Shirasaka, Tomoo Yoshie, Yoshinori Munetomo, Yoshio Sakamoto, Tatsuya Osuga, Saori Matsui, Toshiki Hyodo, Tamami Denda, Daisuke Watanabe, Makoto Ooi, Yuzo Kodama

**Affiliations:** 1 Division of Gastroenterology, Department of Internal Medicine, Kobe University Graduate School of Medicine, 7-5-1 Kusunoki-cho, Chuo-ku, Kobe, 650-0017 Japan; 2 Division of Gastroenterology, Akashi Medical Center, Akashi, Japan; 3 Division of Gastroenterology, Konan Medical Center, Kobe, Japan; 4 Division of General Internal Medicine, Hyogo Prefectural Harima-Himeji General Medical Center, Himeji, Japan; 5 Division of Gastroenterology, Japanese Red Cross Kobe Hospital, Kobe, Japan; 6 Division of Gastroenterology, Kita-Harima Medical Center, Ono, Japan; 7 Division of General Surgery, Himeji Central Hospital, Himeji, Japan; 8 Division of Gastroenterology, Hyogo Prefectural Kakogawa Medical Center, Kakogawa, Japan; 9 Division of Gastroenterology, Takatsuki General Hospital, Takatsuki, Japan; 10 Division of Gastroenterology, Yodogawa Christian Hospital, Osaka, Japan; 11 Department of Diagnostic Pathology, Kobe University Graduate School of Medicine, Kobe, Japan; 12 Department of Pathology, The Institute of Medical Science Research Hospital, The University of Tokyo, Tokyo, Japan

**Keywords:** Biomarkers, Gastroenterology

## Abstract

Not all patients with ulcerative colitis (UC) respond initially to treatment with biologic agents, and predicting their efficacy prior to treatment is difficult. Vedolizumab, a humanized monoclonal antibody against alpha 4 beta 7 (α4β7) integrin, suppresses immune cell migration by blocking the interaction between α4β7 integrin and mucosal addressin cell adhesion molecule 1. Reports about histological features that predict vedolizumab efficacy are scarce. So, we examined the association between histological features and vedolizumab efficacy. This was a multicenter, retrospective study of patients with UC treated with vedolizumab. Biopsy specimens taken from the colonic mucosa prior to vedolizumab induction were used, and the areas positively stained for CD4, CD68, and CD45 were calculated. Clinical and histological features were compared between those with and without remission at week 22, and the factors associated with clinical outcomes were identified. We enrolled 42 patients. Patients with a high CD4+ infiltration showed a better response to vedolizumab [odds ratio (OR) = 1.44, *P* = 0.014]. The concomitant use of corticosteroids and high Mayo scores had a negative association with the vedolizumab response (OR = 0.11, *P* = 0.008 and OR = 0.50, *P* = 0.009, respectively). Histological evaluation for CD4+ cell infiltration may be helpful in selecting patients who can benefit from vedolizumab.

## Introduction

Ulcerative colitis (UC) is a chronic inflammatory disease involving the colonic mucosa. Although the mechanism of the disease is not fully understood, several factors, such as genetic and environmental factors, are speculated to be important triggers for disease onset^1^. UC has no cure, but various treatments can reduce the inflammation^2,3^. As the knowledge of pathological immune cell activation in UC has accumulated, several biologic drugs targeting aberrantly activated immune responses, such as anti-tumor necrosis factor (anti-TNF) and anti-interleukin-12/23 p40 agents, have been approved for UC in recent years^4–7^. Their use leads to the induction and maintenance of the clinical response and clinical remission, even in patients who had a poor response to conventional therapy. However, not all patients respond to each biologic agent, and predicting the efficacy of these agents before treatment initiation is difficult. Therefore, patients often have to start treatment without knowing whether they will respond to a specific agent, and if an adequate response is not achieved, switching to another agent is suggested to successfully induce remission. This could expose patients to the risk of unnecessary adverse effects, in addition to several days or weeks of uncontrolled active UC, before finding an effective treatment. Therefore, using predictors of the primary response to each agent would be beneficial.

Vedolizumab, which is one of the biologic drugs for patients with UC, is a humanized IgG1 monoclonal antibody against alpha 4 beta 7 (α4β7) integrin that blocks gut lymphocyte trafficking by inhibiting the interaction between α4β7 integrin and mucosal addressin cell adhesion molecule-1^8^. Vedolizumab was reported to be effective in patients with UC with moderate-to-severe disease activity; however, not all patients benefit from vedolizumab. A phase 3 trial demonstrated that 47.1% of patients with UC achieved clinical response at week 6 and that 41.8% of responders were in clinical remission at week 52^9^. Real-world data also show that the clinical response rate is 43–57% and that the clinical remission rate is 28–39% at week 14^10,11^. Vedolizumab, as well as other treatments, is not effective in all patients, and predicting which patients can benefit from vedolizumab is difficult. Several studies have described predictors of the efficacy of vedolizumab^12^, but reports about the predictors using histological features are scarce^13^. Specifically, no reports have mentioned an association between a particular phenotype of immune cells and vedolizumab efficacy. Both innate and adaptive immune cells, such as macrophages and CD4+ helper T cells, are involved in pathogenesis in the colonic mucosa of patients with UC^14^. Given the mechanism of action of vedolizumab, we hypothesized that there is an association between vedolizumab efficacy and histological findings prior to vedolizumab initiation. In this study, we aimed to evaluate the efficacy of vedolizumab and identify the factors associated with remission with vedolizumab, including histological features.

## Results

A total of 42 patients were included in this study. The baseline characteristics are summarized in Table 1. The median age was 41.5 years, and 16 (38.1%) patients were male. The median age at diagnosis was 29.5 years, and 27 (64.3%) patients had extensive colitis. With regard to disease activity, 41 (97.6%) and 31 (73.8%) patients had moderate clinical and moderate endoscopic activities, respectively. Systemic corticosteroids were concomitantly used in 16 (38.1%) patients. Among the 42 patients, 19 (45.2%) had a prior use of biologics.

**Table 1:** Baseline characteristic of the patients.

Baseline characteristics (n = 42)	Median (IQR) or number (%)
Age (years)	41.5 (17–85)
Male sex	16 (38.1)
Age at diagnosis (years)	29.5 (10–83)
Duration of disease (years)	6 (0–37)
Smoking	6 (16.7)
Duration between endoscopy and induction of vedolizumab (days)	45 (0–274)
Colonic area involved	
Proctitis	2 (4.8)
Left-sided colitis	13 (31.0)
Extensive colitis	27 (64.3)
Mayo score: 6–10/11–12	41 (97.6)/1 (2.4)
MES: 2/3	31 (73.8)/11 (26.2)
C-reactive protein (mg/L)	2.9 (0.78–48.9)
Hemoglobin concentration (g/dL)	11.85 (6.1–16.4)
Concomitant medication	
Systemic corticosteroids	16 (38.1)
5-aminosalicylates	11 (26.2)
Immunosuppressants	12 (28.6)
Prior use of biologics	19 (45.2)

*IQR* Interquartile range, *MES* Mayo endoscopic subscore.

### Efficacy of vedolizumab

**Figure 1:**
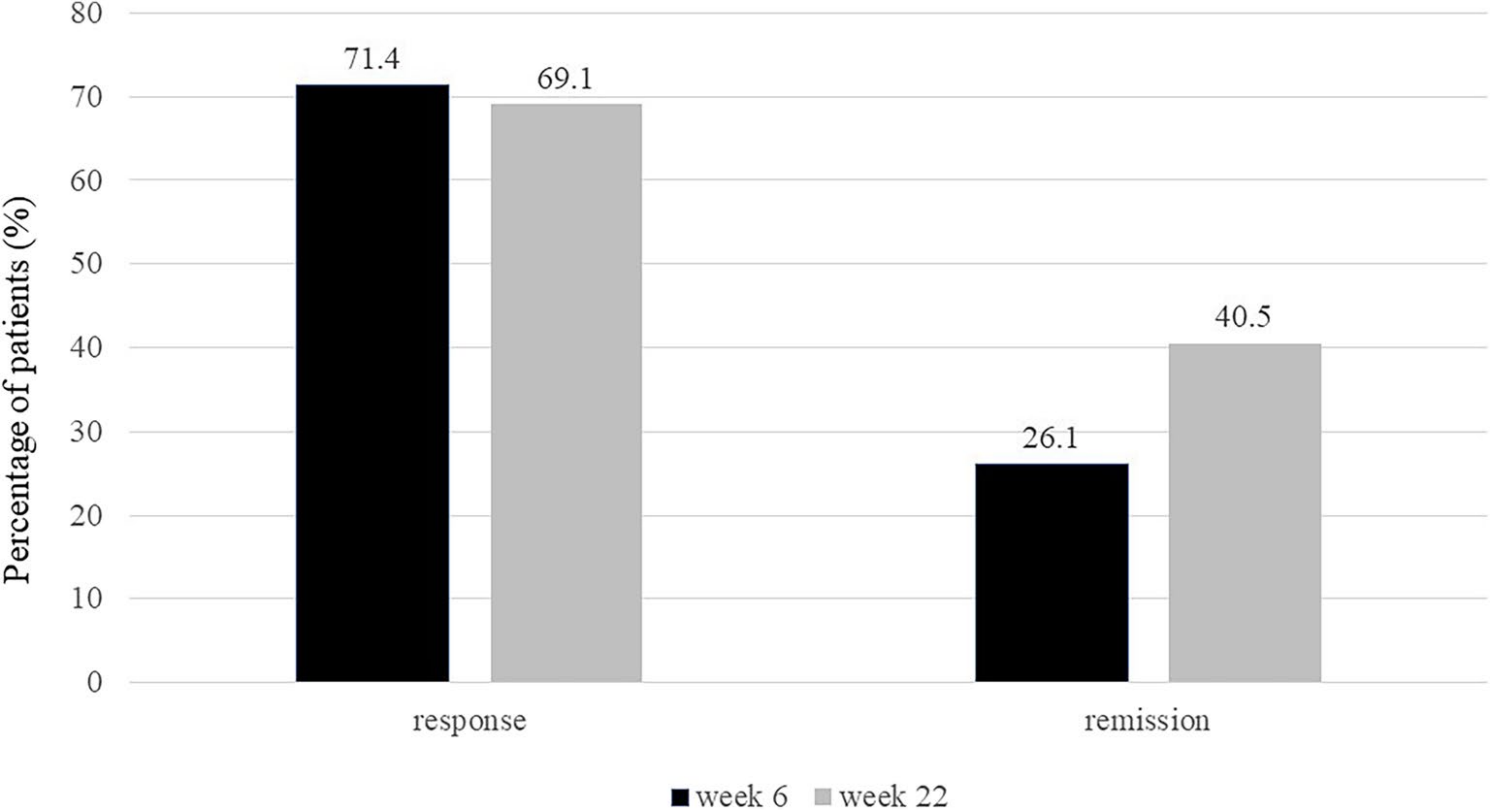
Efficacy of vedolizumab. Graph shows the clinical response rate and clinical remission rate at weeks 6 and 22.

Thirty (71.4%) and 29 (69.1%) patients achieved a clinical response at weeks 6 and 22, respectively. In addition, 11 (26.1%) and 17 (40.5%) patients achieved clinical remission at weeks 6 and 22, respectively (Fig. 1). A total of 34 (81.0%) patients were still receiving vedolizumab at week 22.

### Demographic profiles of patients with and without remission at week 22

In the univariate analysis for remission, no statistical differences were found in age (≥ 40 years), sex, age at diagnosis (≥ 40 years), duration of disease (≥ 10 years), smoking status, colonic area involved (extensive colitis), MES, CRP, hemoglobin, concomitant medication, and prior use of biologics between patients with remission and those without remission at week 22 (Table 2). The Mayo score and concomitant use of systemic corticosteroids were inversely associated with remission (odds ratio [OR] 0.50; 95% confidence interval [CI] 0.29–0.86, *P* < 0.01; OR 0.11; 95% CI 0.02–0.56, *P* < 0.01, respectively). As for histological disease activity, we evaluated Robarts histopathology index (RHI)^15^ and lymphocytic cryptitis. There was no statistical difference in RHI and lymphocytic cryptitis before the treatment (Table 2).

**Table 2:** Demographic profile of patients with remission (n = 17) and those without remission (n = 25) at week 22.

Characteristic	Remission, n (%)	No remission, n (%)	OR (95% CI)	*P* value
Age ≥ 40 years	6 (35.3)	16 (64)	0.31 (0.08–1.11)	0.072
Male sex	8 (47.1)	8 (32)	1.89 (0.53–6.73)	0.326
Age at diagnosis ≥ 40 years	4 (23.5)	12 (48)	0.33 (0.08–1.31)	0.115
Duration of disease ≥ 10 years	4 (23.5)	11 (44)	0.39 (0.10–1.54)	0.180
Smoking	1 (5.9)	6 (24)	0.20 (0.02–1.82)	0.152
Extensive colitis	8 (47.1)	19 (76)	0.28 (0.07–1.05)	0.060
Mayo score*	7.29	8.44	0.50 (0.29–0.86)	0.009
MES*	2.12	2.36	0.24 (0.04–1.28)	0.086
CRP (mg/dL)*	0.83 ± 0.10	2.90 ± 2.98	0.64 (0.30–1.37)	0.249
Hemoglobin concentration (g/dL)*	13.6 ± 1.18	11.9 ± 2.35	1.33 (0.98–1.81)	0.071
Concomitant medication				
Systemic corticosteroids	2 (11.8)	14 (56)	0.11 (0.02–0.56)	0.008
≥ 20 mg/day	1 (5.9)	5 (20)	0.25 (0.03–2.36)	0.226
5-Aminosalicylates	6 (35.3)	5 (20)	2.18 (0.54–8.82)	0.273
Immunosuppressants	3 (17.6)	9 (36)	0.38 (0.09–1.69)	0.204
Prior use of biologics	9 (52.9)	10 (40)	1.69 (0.49–5.85)	0.410
RHI*,**	15.1 ± 7.2	15.0 ± 9.3	1.00 (0.93–1.08)	0.975
Lymphocytic cryptitis*,**	1.31 ± 0.48	1.17 ± 0.64	1.58 (0.51–4.91)	0.431

*OR* Odds ratio, *CI* Confidence interval, *MES* Mayo endoscopic subscore, *RHI* Robart histopathology index.

*Continuous variables are summarized as mean and standard deviation.

**Two samples which did not have epithelium were omitted from the analysis (total of 40 samples were evaluated).

### Evaluation of immune cell infiltration in colonic tissue by immunohistochemistry

There are many types of innate and adaptive immune cells involved in the pathology of UC, and CD4 was one of the markers selected for staining. CD4-positive helper T cells are well-known effector cells of adaptive immunity and good targets of vedolizumab, because they are one of the major cell populations recruited into intestinal mucosa using α4β7 integrin. For comparison, we selected CD68, a monocyte/macrophage marker, for the innate cell population. Monocytes can turn into macrophage once recruited to the peripheral tissue, and monocytes express significantly low levels of α4β7 integrin^16^, which suggests that vedolizumab may minimally affect monocyte/macrophage recruitment into the intestines.

**Figure 2:**
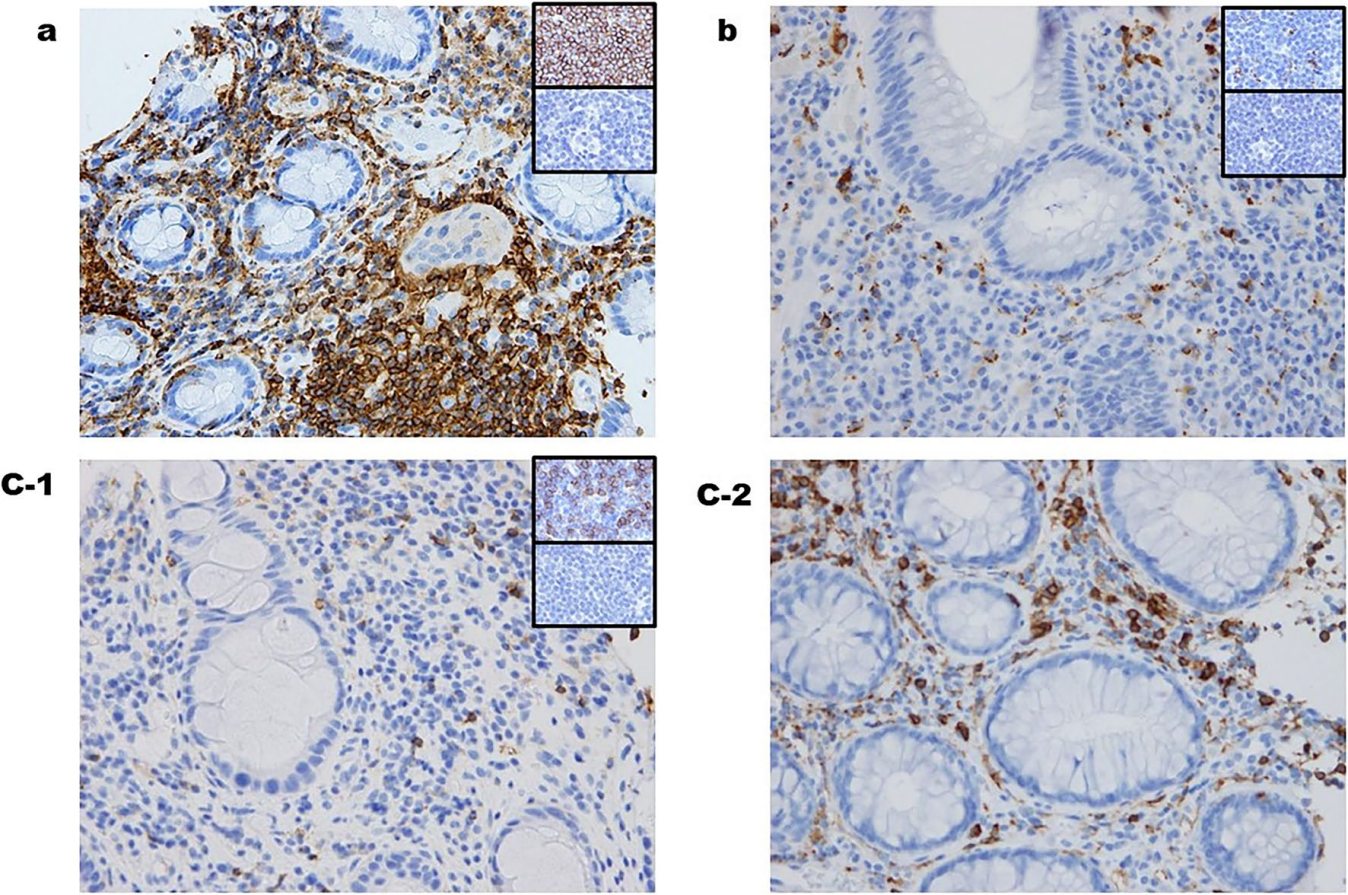
Representative images of immunohistochemical staining for CD45 (**a**), CD68 (**b**), and CD4 (**c-1** shows a case of mild CD4 infiltration and **c-2** shows a case of severe CD4 infiltration) with positive (upper right, top) and negative (upper right, bottom) staining controls.

**Figure 3:**
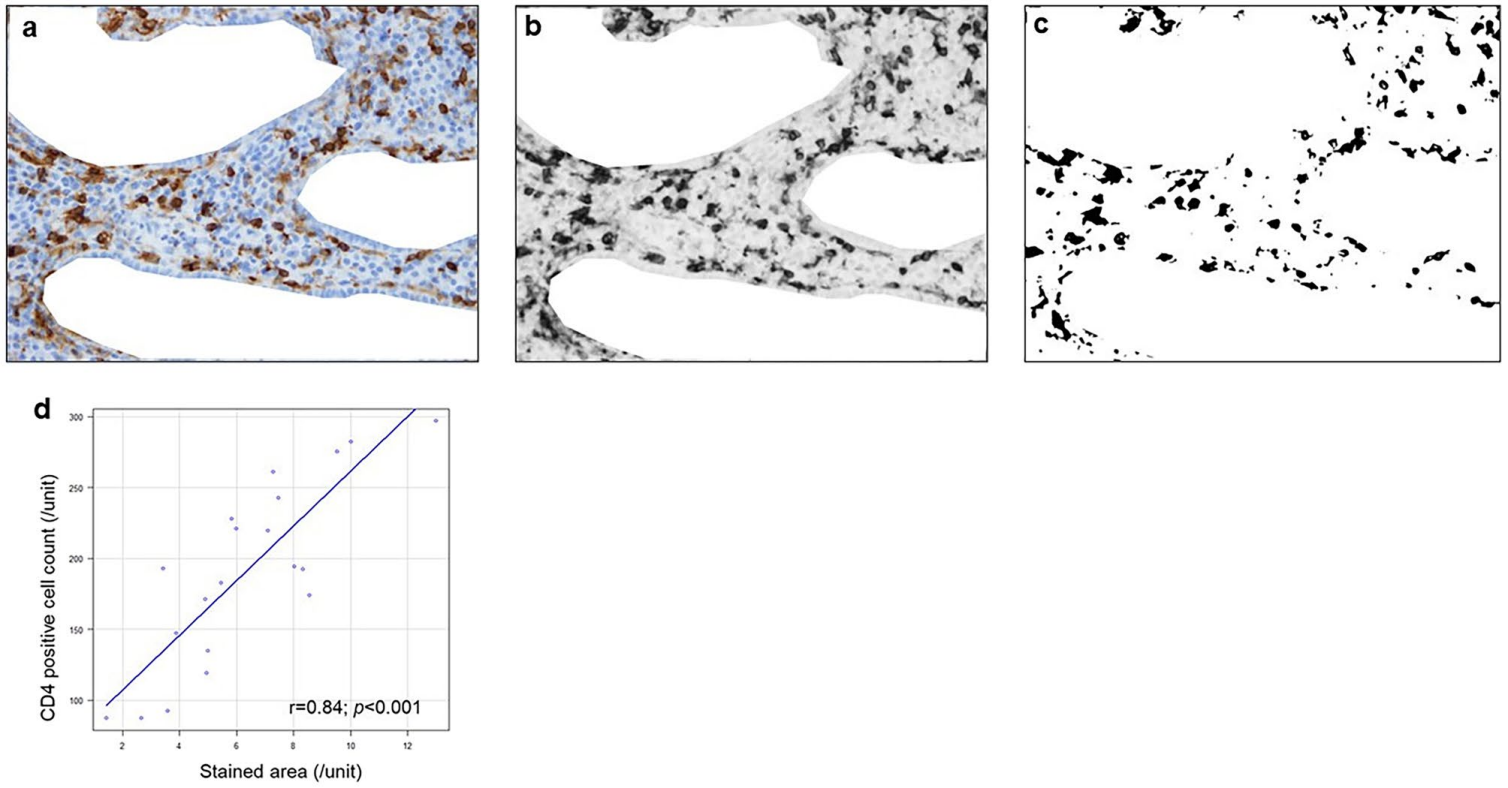
Representative images processed by image J software for the quantification of CD4 staining (**a**–**c**). Confirmation of the correlation between the stained area calculated by image J software and the manually counted positive cell number using 20 representative samples. The Pearson correlation was used to assess the correlation between the stained area calculated by Image J and the cell count (**d**).

Regarding the histological evaluation, clearly positive staining for CD45, CD4, and CD68 with very low background signal was achieved (Fig. 2). The quantification of the positive-cell-stained area correlated well with the manually counted infiltrating immune cell number (r = 0.84;* P* < 0.001) (Fig. 3).

The CD45-positive area was very similar between the remission and non-remission groups, indicating similar levels of immune cell infiltration in both groups before vedolizumab induction which may be supported by the similar RHI mentioned above. However, significantly more CD4+ cells were found in the colonic mucosal tissues of patients with remission than in those without remission (OR 1.44; 95% CI 1.08–1.92, *P* = 0.014) (Table 3). The median value of proportion of CD4-positive cells among CD45-positive cells was 24.6% (range: 4.60%-89.9%), and the proportion of CD4-positive cells among CD45-positive cells had tendency to be higher in the remission group than in the non-remission group (31.9% vs 20.3%, *P* = 0.066). In contrast, the CD68-positive area was not significantly different between the two groups (*P* = 0.904).

**Table 3:** Relationship between remission at week 22 and expression of CD4, CD68, and CD45 in the colonic mucosa.

Characteristic	OR (95% CI)	*P* value
CD4-positive area	1.44 (1.08–1.92)	0.014
CD68-positive area	0.96 (0.51–1.81)	0.904
CD45-positive area	1.01 (0.96–1.07)	0.702
Proportion of CD4 cells among CD45 cells	20.9 (0.81–536)	0.066

*OR* Odds ratio, *CI* Confidence interval.

**Figure 4:**
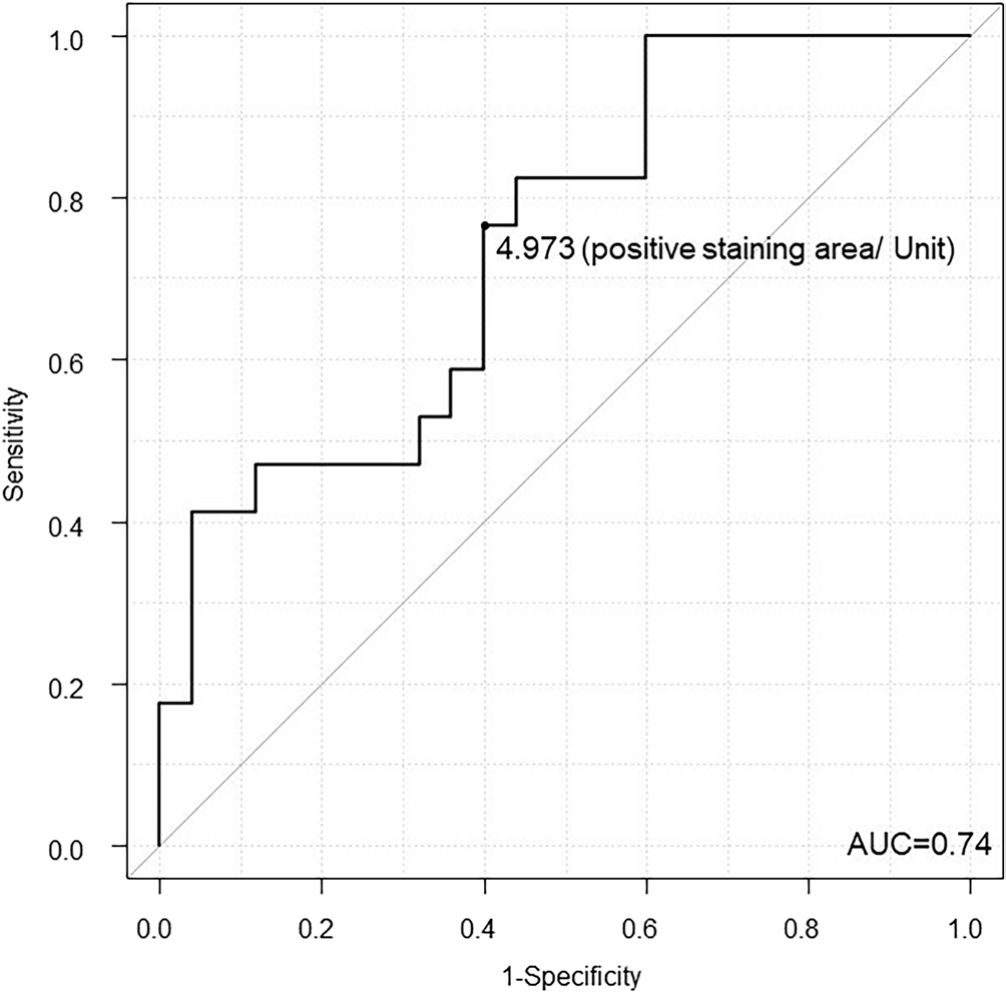
Receiver operating characteristic curve of the CD4-positive area. *AUC* Area under the curve.

Figure 4 shows the ROC curve for CD4+ infiltration in predicting remission. The ROC showed that the AUC was 0.741, and the optimal cutoff value was 4.973 (positive staining area/unit), providing a sensitivity of 76.5% and a specificity of 60.0%.

### Multivariate analysis for predicting responder

In order to identify independent predictors of responder at week 22, we conducted a multivariate logistic regression analysis with the Mayo score, concomitant use of systemic corticosteroid, and positive area of CD4. The results showed that positive area of CD4 was the independent predictor (*P* < 0.05) (Table 4).

**Table 4:** Multivariate logistic regression analysis for predicting factor associated with remission at week 22.

Characteristic	OR (95% CI)	*P* value
Mayo score	0.45 (0.23–0.89)	0.021
Concomitant use of corticosteroid	0.11 (0.02–0.74)	0.023
Positive area of CD4	1.5 (1.03–2.18)	0.034

*OR* Odds ratio, *CI* Confidence interval.

## Discussion

In this study, we found an association between the response to vedolizumab and the following factors: CD4+ cell infiltration in the colonic mucosa, non-concomitant use of systemic corticosteroids, and mild disease activity. As we hypothesized, the efficacy of vedolizumab was associated with the histological features prior to the vedolizumab initiation. Some studies have suggested predictors of the response to vedolizumab. Narula et al*.* showed that the previous use of anti-TNF agents is a risk factor for failure of treatment with vedolizumab^16^. They also reported that proctitis (versus more extensive disease) is a statistically significant predictor of the clinical response. Regarding the disease severity, some studies showed that patients with mild disease activity seem to benefit more from vedolizumab than those with severe disease activity^10,11,17^. Several studies reported an association between low CRP levels and a better response to vedolizumab^10,18^. Patients with severe disease activity were likely to show a poor response to vedolizumab; accordingly, our results also detected a higher Mayo score as a factor for a poor response. In patients with UC, the tissue levels of TNFα have been shown to correlate with the severity of the disease^19^. Considering the mechanism of action of vedolizumab, completely blocking severe inflammation with high TNFα levels might be difficult. Nevertheless, the fact that vedolizumab is effective in some severe colitis cases suggests that there are mechanisms by which these different responses exist. One mechanism can be attributed to differences in the immunological phenotypes of each patient. Inflammatory bowel disease can be classified into subphenotypes based on the inflammatory protein profiles^20^, and mucosal and systemic immune profiles can be altered in the course of the disease^21^. This suggests that understanding the immunopathology of each patient may help to guide the individualized selection of biologics.

In the present study, a high infiltration of CD4+ cells in the colonic mucosa was associated with a good response to vedolizumab. Although α4β7 integrin is expressed on several subsets of leukocytes, it is preferentially expressed on CD4+ T cells^16^. This may explain why patients with a high infiltration of CD4+ T cells in the colonic mucosa benefit more from vedolizumab than those with a low infiltration of CD4+ T cells. In addition to CD4+ T cells, multiple inflammatory cell types contribute to the pathogenesis of UC^22^. For example, innate lymphoid cells (ILCs), which lack antigen-specific receptors, were identified as innate immune cells, but they can produce cytokines and mediate immunity, homeostasis, and inflammation. ILCs are classified as ILC1, ILC2, and ILC3 according to their cytokine profiles and development^23^. ILC1s share the biological activity of T helper 1 cells and can produce interferon-γ and TNF^24^. ILC1 levels have been reported to increase in the inflamed gut tissue of patients with inflammatory bowel disease when compared with those in healthy controls^25^, which indicates that ILC1s may also be involved in the pathogenesis of inflammatory bowel disease. Given that ILCs are CD4-negative cells and the expression of α4β7 integrin on ILCs in the mucosa of patients with inflammatory bowel disease is low^25^, ILCs may play a central role in the pathogenesis of UC in patients who show a poor response to vedolizumab.

Corticosteroids inhibit the expression of mucosal addressin cell adhesion molecule-1 in endothelial cells^26^. This leads to the inhibition of lymphocyte binding to the endothelium^27^, as with vedolizumab. This may be the reason why the concomitant use of systemic corticosteroids with vedolizumab showed a negative association with remission in the present study.

Finally, the ROC analysis of the CD4-positive area used to differentiate between patients with remission and those without remission at week 22 showed an AUC of 74%, suggesting moderate accuracy. This study involved staining for only three markers, CD4, CD68, and CD45, partly because simplicity is important for daily use. However, improving the accuracy should be investigated; moreover, analyzing the staining information of other cell types involved in the UC pathology, such as B cells and ILCs, and analyzing the data with respect to the current results would be interesting.

Our study had several strengths. To our knowledge, this is the first study to report that a high infiltration of CD4+ cells in the colonic mucosa may lead to a good response to vedolizumab treatment. Additionally, we sought to minimize the staining variation among specimens by performing immunostaining at a single facility.

As this study was retrospective and exploratory, it also had several limitations. First, although several medical centers were involved, the sample size was small. A prospective study with a larger sample size from baseline throughout the course of treatment with vedolizumab is warranted. Second, we did not precisely evaluate which infiltrating cells express α4β7 integrin, which could provide more direct information regarding the mode of action of vedolizumab. Third, owing to the retrospective nature of the study, we could not obtain biopsy samples at week 22 to confirm whether vedolizumab blocked CD4+ cell recruitment in the remission group. The duration of biopsy sample taken before initiation of vedolizumab therapy was not strictly controlled, and spanned relatively long period (median 45 days).

In conclusion, we found an association between CD4+ cell infiltration in the inflamed colonic mucosa and the response to vedolizumab. Given that various novel therapeutics have emerged that they do not work for everyone, choosing a therapy with the highest response for each patient is important. Our results suggest that the histological evaluation of CD4+ cell infiltration may be helpful for the selection of patients who can benefit from vedolizumab.

## Methods

### Study design

This retrospective multicenter study was conducted in nine hospitals (Akashi Medical Center, Hyogo Prefectural Kakogawa Medical Center, Kita-Harima Medical Center, Konan Medical Center, Japanese Red Cross Kobe Hospital, Takatsuki General Hospital, Himeji Central Hospital, Yodogawa Christian Hospital, and Kobe University Hospital) in Japan. Participating institutions included both city and university hospitals, to reduce the patient deviations.

Patients with UC who were treated with vedolizumab between November 2018 and April 2021 were enrolled in this study. All eligible patients were diagnosed with UC on the basis of the Japanese Diagnostic Criteria for UC^28^. They had moderate-to-severe disease activity at the time of the initial treatment with vedolizumab, defined as a full Mayo score of ≥ 6 and a Mayo endoscopic subscore (MES) of ≥ 2^29^. In the analysis, we used the biopsy specimen obtained from the most inflamed area of the colon during colonoscopy within 12 months prior to the initial treatment of vedolizumab. Vedolizumab was intravenously administered at a standard dosing regimen (300 mg at weeks 0, 2, and 6, and then every 8 weeks thereafter).

Data, including patient characteristics (age, sex, age at diagnosis, duration of disease, and smoking status), disease characteristics (colonic area involved, Mayo score, MES, C-reactive protein [CRP], and hemoglobin), and treatment history (concomitant medication and prior use of biologics), were collected.

### Study outcomes

The primary outcome was the clinical remission rate at week 22. Clinical remission was defined as a partial Mayo score (PMS; Mayo score without endoscopic subscore) of ≤ 1 and a rectal bleeding score of 0 without additional treatment. The secondary outcomes were the clinical remission rate at week 6, clinical response rate at weeks 6 and 22, and factors associated with clinical remission at week 22. A clinical response was defined as a reduction in the PMS of at least 2 points and a decrease of at least 25% from the baseline score, with a decrease of at least 1 point in the rectal bleeding subscore or an absolute rectal bleeding subscore of 0 or 1 without additional treatment. The factors that we explored included the basic characteristics of the patients and their histological features. Histological features were evaluated using immunohistochemistry for CD45 (a marker of hematopoietic cells, except for red blood cells and platelets), CD4 (a marker of T helper cells), and CD68 (a macrophage marker). Histological disease activities were also evaluated using Robarts histopathology index (RHI)^15^ and the presence of lymphocytic cryptitis (3-point scale; 0 = none, 1 = mild, 2 = moderate or severe). The scoring was done by a single pathologist blinded from the information of the patients.

### Immunohistochemistry

In the analysis, we used a biopsy sample obtained from the most inflamed area of the colon within 12 months prior to vedolizumab initiation. Formalin-fixed, paraffin-embedded, 3-μm-thick tissue sections were deparaffinized, and the endogenous peroxidase activity was blocked by incubation with 3% hydrogen peroxidase for 5 min at 25 °C. Antigen retrieval was performed using 10 mM citrate buffer (pH 6.0; Vector Laboratories, Burlingame, CA, USA) for 10 min at 120 °C in a pressure cooker (Panasonic, Osaka, Japan). The sections were then incubated for 30 min at 37 °C with the following primary antibodies: CD4 (clone EPR6855, dilution 1:250, ab133616; Abcam, Cambridge, UK), CD45 (clone D9M8I, dilution 1:300, #13917; Cell Signaling Technology), and CD68 (clone PG-M1, dilution 1:200, M0876; Agilent Technologies, Santa Clara, UK). For the negative control, the following isotype controls were used at the same concentration to the primary antibodies of interests: CD4 (Rabbit IgG isotype control, clone EPR25A, ab172730, Abcam; CD45 (Rabbit IgG isotype control, clone EPR25A, ab172730, Abcam; CD68 (Mouse IgG3 kappa isotype control, clone MG3-35, ab18394, Abcam). After washing with Tris-buffered saline, the sections were treated with a secondary antibody (Envision + Dual Link System-HRP, K4061; Agilent Technologies) for 30 min at 37 °C. To visualize the antigen–antibody complex, ImmPACT DAB substrate peroxidase (SK-4105) was used, and the sections were counterstained with hematoxylin. Normal lymph nodes were placed on the same glass slide as a positive control, and negative controls were stained with phosphate-buffered saline instead of the primary antibodies.

To objectively evaluate the level of positively stained cell infiltration, we quantified the stained area using image analysis software (Image J software, version 1.52; US National Institutes of Health, Bethesda, Maryland, USA). Two investigators blinded from the biopsy information randomly picked area including lamina propria to take pictures. For each biopsy specimen, three consecutive non-overlapping microscopic fields were identified and captured. The total area of the lamina propria was measured after the removal of the cryptic lumen and epithelium. Areas of positive staining for CD4, CD45, and CD68 were also measured. The area of positively stained cells of CD4, CD45, and CD68 per unit area of the lamina propria was calculated by dividing the positive area by the area of the total lamina propria. Measurements were performed at 400 × magnification.

### Statistical analysis

Continuous variables are summarized using medians and interquartile ranges. Categorical variables are expressed as numbers and percentages. For intergroup comparisons, the Mann–Whitney U test was used to compare continuous variables, and the Pearson chi-square test was used to compare binary variables. Pearson correlation was used to examine the relationship. Univariate and multivariate logistic analysis was performed to determine the independent markers for predicting responder. Receiver operating characteristic (ROC) analysis was performed to calculate the area under the receiver operating characteristic curve (AUC) for the performance of positive staining of CD4 to distinguish responders from non-responders. All P-values were two-tailed, and statistical significance was set at *P* ≤ 0.05. Statistical analyses were performed using EZR software package (version 1.40; Saitama Medical Center, Jichi Medical University, Saitama, Japan)^30^.

### Ethical considerations

The requirement for written informed consent to participate in the study was replaced by an opt-out method, owing to the retrospective nature of this study. The institutional review board (the Ethics Committee of Kobe University Hospital) approved this study and waived informed consent (approval number: B200092). All methods were performed in accordance with the principles of the Declaration of Helsinki and institutional regulations and guidelines.

## Data Availability

The data that support the findings of this study are available on request from the corresponding author, NH. The data are not publicly available due to their containing information that could compromise the privacy of research participants.
